# Activated *i*NKT Cells Promote Memory CD8^+^ T Cell Differentiation during Viral Infection

**DOI:** 10.1371/journal.pone.0037991

**Published:** 2012-05-23

**Authors:** Emma C. Reilly, Elizabeth A. Thompson, Sandrine Aspeslagh, Jack R. Wands, Dirk Elewaut, Laurent Brossay

**Affiliations:** 1 Division of Biology and Medicine, Department of Molecular Microbiology and Immunology and Graduate Program in Pathobiology, Brown University, Providence, Rhode Island, United States of America; 2 Laboratory for Molecular Immunology and Inflammation, Department of Rheumatology, Faculty of Medicine and Health Sciences, Ghent University, Ghent, Belgium; Karolinska Institutet, Sweden

## Abstract

α-galactosylceramide (α-GalCer) is the prototypical lipid ligand for invariant NKT cells. Recent studies have proposed that α-GalCer is an effective adjuvant in vaccination against a range of immune challenges, however its mechanism of action has not been completely elucidated. A variety of delivery methods have been examined including pulsing dendritic cells with α-GalCer to optimize the potential of α-GalCer. These methods are currently being used in a variety of clinical trials in patients with advanced cancer but cannot be used in the context of vaccine development against pathogens due to their complexity. Using a simple delivery method, we evaluated α-GalCer adjuvant properties, using the mouse model for cytomegalovirus (MCMV). We measured several key parameters of the immune response to MCMV, including inflammation, effector, and central memory CD8^+^ T cell responses. We found that α-GalCer injection at the time of the infection decreases viral titers, alters the kinetics of the inflammatory response, and promotes both increased frequencies and numbers of virus-specific memory CD8^+^ T cells. Overall, our data suggest that iNKT cell activation by α-GalCer promotes the development of long-term protective immunity through increased fitness of central memory CD8^+^ T cells, as a consequence of reduced inflammation.

## Introduction

Adjuvants are mediators that enhance the natural immune response. Two vaccine adjuvants are approved in the United States for prophylactic vaccination; aluminum adjuvants (Alum) and monophosphoryl lipid A (MPLA). Alum is currently used to boost immune responses in conjunction with a number of vaccines including those against hepatitis A, tetanus, and influenza, while MPLA (a derivative of Salmonella minnesota LPS) is currently used as an adjuvant for the human papillomavirus vaccine [1]. A major downfall of these adjuvants, however, is that they do not effectively promote protective cell-mediated immunity [2]. Cell-mediated immunity marked by robust CD8^+^ T cell responses is critical for developing efficacious vaccines against diseases such as malaria and human immunodeficiency virus. Previous attempts to generate vaccines against a variety of diseases including HIV, malaria and tuberculosis have been mostly unsuccessful. To prevent these infections, it is believed that vaccines will need to induce the generation of an adequate and strong CD8^+^ T cell memory response [3]. Immunological memory is essential for protection from previously encountered pathogens and can limit reactivation of existing latent infections [4]. CD8^+^ memory T cells respond efficiently and robustly, mounting a specific response much faster than their naïve counterpart [5]. Memory T cells show directed cytokine production, long term survival, and an ability to self renew. Recently, using a variety of cell surface markers, several groups have been able to distinguish memory precursor effector cells (MPECs) as CD8^+^ T cells that have a potential to survive and become long-lived memory CD8^+^ T cells from short-lived effector cells (SLECs) [6], [7].

Recent studies have evaluated α-Galactosylceramide (α-GalCer) as a potential adjuvant due to its ability to induce the activation of a variety of immune cells [8], [9], [10], [11], [12], although in at least one case α-GalCer treatment fails to control viral replication [13]. α-GalCer is the powerful iNKT agonist and is presented by the nonclassical MHC molecule CD1d in both mice and humans [14]. α-GalCer administrated with a variety of vaccines increases their efficacy and its action is mediated in part by IFN-γ [15].

In the case of CD8^+^ T cell responses, treatment with α-GalCer has been shown to increase the CD8^+^ memory T cell population in the context of an influenza vaccine through upregulation of the prosurvival gene Bcl-2, in mice [16]. The evidence that α-GalCer has an effect on long term CD8^+^ T cell memory is limited in other viral systems and its mechanism of action on the generation of MPECs versus SLECs is unknown. We therefore sought to determine the role and mechanism of α-GalCer during murine cytomegalovirus (MCMV) infection. MCMV is the model for the human β-herpesvirus HCMV and presents with pathologically similar features to the human form of the virus [17]. CMV induces a strong acute response marked by NK cell cytotoxicity as well as cytolytic CD8^+^ T cell activity [18], [19]. Following acute infection, the virus migrates from the primary organs of infection to the salivary glands in both mice and humans where it remains for up to a few months [20], [21]. After this point, the virus becomes virtually undetectable in both systems. This latent form however, can reactivate upon an immunocompromised state and pose serious health risks such as retinitis, colitis, or liver damage. An optimal CD8^+^ memory T cell response is therefore critical to maintaining latency of the virus.

Here, we show that a single α-GalCer injection at the time of the infection decreases viral titers, alters the kinetics of the inflammatory response, and promotes both increased frequencies and numbers of virus-specific memory CD8^+^ T cells. We present a model in which adequate iNKT cell activation could potentially promote long-term immunity and help prevent viral reactivation.

## Results

### α-GalCer treatment interferes with MCMV induced cytokine production

**Figure 1:**
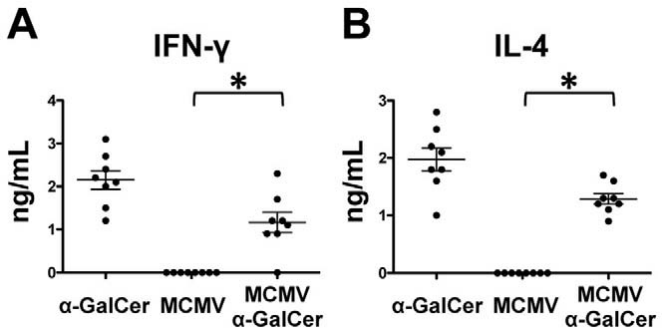
α-GalCer promotes Th1 and Th2 cytokine production by 4 hours. IFN-γ (A) and IL-4 (B) levels were determined at 4 hours in blood serum after α-GalCer treatment and/or MCMV infection by ELISA. *p-value<0.005.

**Figure 2:**
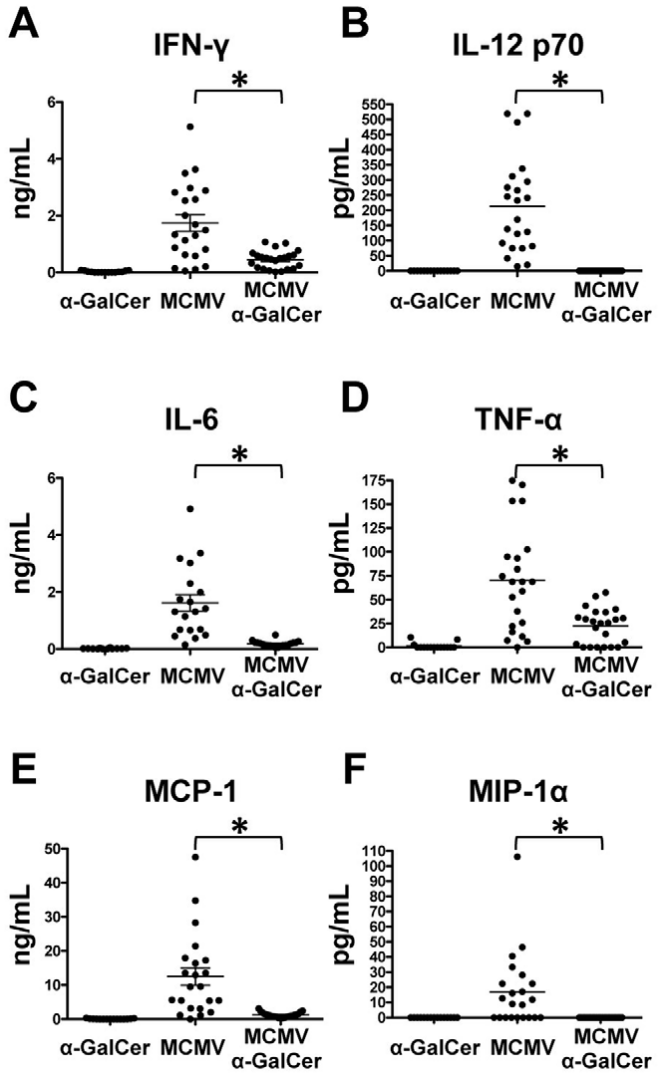
α-GalCer treatment modulates MCMV-dependent cytokine profile. Blood sera were collected at 38 hours post-infection and cytokines measured using the BD CBA Flex Kit. Cytokines not detected under any condition tested: IL-10, IL-4, IL-21, IL-17A, GM-CSF. *p-value≤0.002.

α-GalCer treatment results in rapid production of IFN-γ and IL-4, as well as other cytokines in the serum at 4 hours post-treatment as a consequence of direct iNKT cell activation (Fig. 1 and data not shown). MCMV replication promotes production of pro-inflammatory cytokines and chemokines (IL-12p70, IFN-γ, TNF-α, MIP-1α, IL-6, and MCP-1), which peak between 24 and 40 hours post-infection in the blood depending on the cellular source of the cytokine [22]. At 38 hours post-infection with no α-GalCer treatment, IFN-γ reaches its peak and IL-12p70 is detectable in the blood (Fig. 2). However, IL-12p70 is absent and serum IFN-γ is severely diminished when α-GalCer is administered in conjunction with MCMV infection (Fig. 2). Similarly, TNF-α, MCP-1, IL-6, and MIP-1α responses are all decreased at this time point after α-GalCer treatment (Fig. 2). Notably, IL-10, IL-4, IL-21, IL-17A, GM-CSF are not detected under either of the tested conditions. Thus α-GalCer treatment alters the kinetics of the normal MCMV inflammatory cytokine response.

### Viral titers are severely diminished at 48 hours after α-GalCer treatment

**Figure 3:**
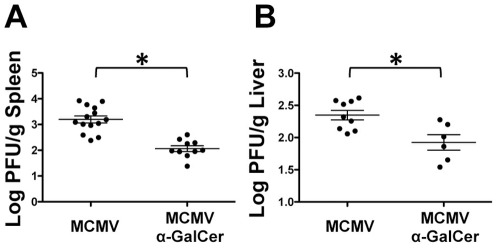
α-GalCer treatment decreases viral titers in the spleen and liver. Spleen (A) and liver (B) were harvested at 48 hr post-infection and MCMV titers were determined by standard plaque assay. *p-value≤0.007.

One of the early studies has shown that α-GalCer administration inhibits viral replication in a hepatitis B virus-transgenic model [23]. Within 24 hours of α-GalCer injection, IFN-γ and IFNα/β are detected in the liver of HBV transgenic mice and HBV replication is abolished. α-GalCer treatment has also been shown to affect viral titers for several models of viral infections [24], [25], [26], [27] including MCMV [28]. Based on these previous studies with α-GalCer, we hypothesized that α-GalCer induced cytokine production at early time points and direct anti-viral effects would result in decreased viral titers at 48 hours post-treatment. MCMV titers in homogenates of spleen and liver samples from MCMV infected and MCMV+α-GalCer groups were determined by standard plaque assay. In agreement with others [28], we found that MCMV titers were significantly reduced in mice treated with α-GalCer in both spleen and liver (Fig. 3). Thus the availability of antigen for MCMV specific CD8^+^ T cells is decreased under these conditions.

### CD8^+^ T cell effector numbers are not decreased by α-GalCer

**Figure 4:**
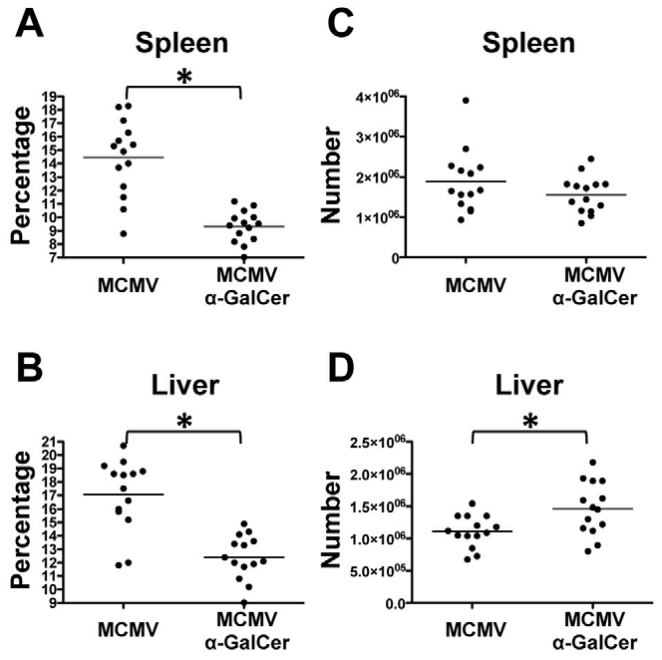
α-GalCer treatment does not decrease effector CD8^+^ T cell population. Cells were stained with a combination of M45 and M57 tetramers at day 7 post-infection. Percentages of tetramer positive cells within CD8^+^ T cell population (A, B). Absolute number of tetramer positive CD8^+^ T cells in each organ (C, D). *p-value≤0.01.

CD8^+^ T cells are major contributors in the acute response to MCMV infection. Despite encoding for approximately 170 proteins and a large number of potential antigens, only several epitopes within MCMV have been described in C57BL/6 mice including M38, M139, M45, and M57 [29]. CD8^+^ T cells specific to M38 and M139 are connected with a population referred to as inflationary cells [30]. In contrast, CD8^+^ T cells specific for other MCMV epitopes (non-inflationary) have been shown to differentiate to a classical central memory phenotype [31]. Here, we focused on classical memory CD8^+^ T cells specific for M45 and M57, which expand during acute infection but have also been associated with long-term protection against MCMV [29]. We first examined the effect of α-GalCer on the effector CD8^+^ T cell response. To do this, the spleens and livers of mice infected with MCMV with and without α-GalCer treatment were evaluated at day 7 post-infection. We utilized a combination of tetramers loaded with M45 or M57 to identify MCMV specific CD8^+^ T cells (representative staining shown in Fig. S1A and B). Despite observing a lower frequency of tetramer positive cells in these organs, there was no overall difference in absolute numbers of MCMV-specific effector CD8^+^ T cells at day 7 in the spleen (Fig. 4) while there was a slight increase in the liver. To further verify that this population maintained its effector status, tetramer positive cells were characterized using the classical markers KLRG1 and CD62L (Fig. S1 C and D). Most tetramer positive cells expressed high levels of KLRG1 and low levels of CD62L, which are indicative of an effector population. Thus, although α-GalCer administration during MCMV infection induces a decreased viral titer the number of antigen specific effector CD8^+^ T cells is not significantly affected.

### Central memory frequency and absolute numbers are increased with α-GalCer

**Figure 5:**
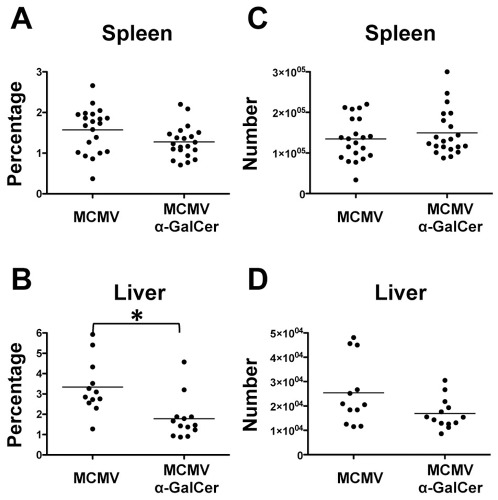
α-GalCer does not alter the overall number of MCMV specific CD8^+^ T cells in the spleen or liver. At 9 weeks post-infection, cells were stained with M45 and M57 tetramers. Frequency of tetramer positive cells in the spleen (A) or liver (B). Absolute number of tetramer positive cells in the spleen (C) or liver (D). p-value = 0.003.

**Figure 6:**
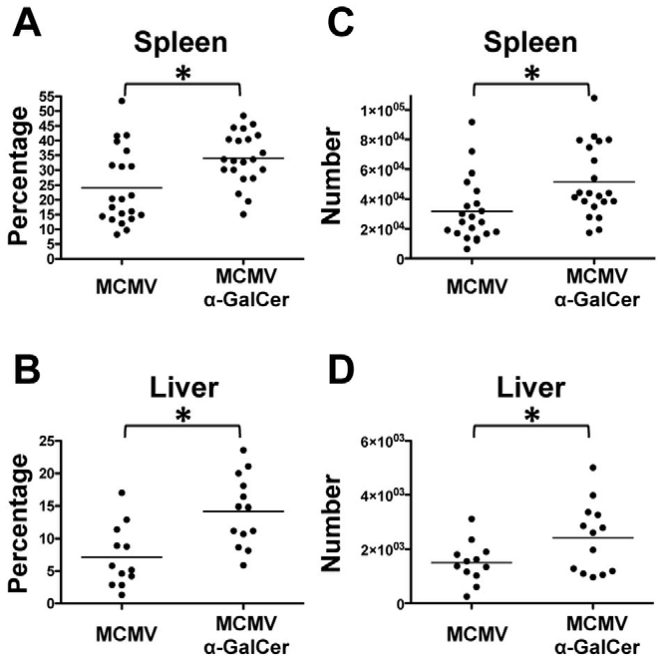
α-GalCer treatment increases the frequency and number of MCMV specific central memory T cells in both spleen and liver. Cells were stained with M45 and M57 tetramers. Frequency of central memory CD8^+^ T cells in spleen (A) and liver (B). Absolute number of MCMV-specific central memory CD8^+^ T cells in both organs (C, D). *p-value<0.05.

It is well known that the early inflammatory milieu dictates the rate at which CD8^+^ T cells acquire memory characteristics [6], [32]. Excess of inflammatory cytokines such as IL-12 and IFN-α has been shown to promote CD8^+^ T cell differentiation leading to a decrease of central CD8^+^ memory T cells [6], [33], [34]. The magnitude of infection, strength of stimulus, and antigen levels can also influence the rate of memory T cell differentiation [35], [36]. Due to the change in the inflammatory cytokine profile and the lower viral titer observed after α-GalCer treatment, we hypothesized that α-GalCer may influence effector versus memory cell fate decision. To define the MCMV-specific central memory response, mice were infected with MCMV with and without α-GalCer treatment and sacrificed 9-weeks later. As anticipated, the frequency of MCMV-specific (defined as M45 tetramer positive or M57 tetramer positive) CD8^+^ T cells drastically decreased since the day 7 time point, as a result of contraction following the acute expansion in response to antigen recognition (Fig. 5A, B). Interestingly, the absolute number of MCMV-specific CD8^+^ T cells in both spleen and liver were comparable between the MCMV+α-GalCer and MCMV control groups (Fig. 5C, D). We further characterized these cells using specific markers for the central memory population. We defined this population as tetramer positive cells that expressed CD62L, CD127, and CD44, but are mostly negative or low for KLRG1 (Fig. S2). Using this phenotype we show that the frequencies of central memory CD8^+^ T cells in MCMV+α-GalCer treated spleen and liver are significantly higher than the frequencies of central memory cells from MCMV infected mice (Fig. 6A, B). Importantly, this phenomenon translates to the absolute number of central memory cells (Fig. 6C, D). These results show that a single α-GalCer treatment at the time of infection promotes accumulation of CD8^+^ central memory T cells specific for MCMV antigens.

## Discussion

Many viruses are controlled by effector CD8^+^ T cells as antibodies are not effective against intracellular pathogens. After disease resolution, these effector cells contract leaving a long-lived central memory population. This CD8^+^ T cell memory population is maintained and expands to prevent infection upon subsequent exposure as well as control chronic or latent infections. Vaccination strategies that elicit an optimal CD8^+^ T cell response directed against intracellular pathogens are actively pursued [37]. Adjuvants are among the factors governing the regulation and differentiation of the CD8^+^ T cell memory response. Here we tested the potential of α-GalCer as an adjuvant during MCMV infection. iNKT cells can respond within minutes to specific glycolipids such as α-GalCer [14], [38] and have the capacity to produce high quantities of both Th1 and Th2 cytokines. This cytokine storm can crosstalk with NK cells or, in conjunction with dendritic cells, promotes activation and responses of both B and T cells [15], [39], [40], [41]. These and other findings led to the development of glycolipid analogs and strategies to polarize iNKT responses [42], [43], [44], [45] to better utilize iNKT properties in the design of adjuvants or vaccines. In contrast to currently used vaccine adjuvants, α-GalCer may be a viable adjuvant to boost cellular responses as it has been show to enhance cellular immune responses to malaria antigens when used as part of a DNA subunit vaccine [8].

A variety of delivery methods have been examined including pulsing dendritic cells with α-GalCer before administration [46] to optimize the potential of α-GalCer. These methods are currently being used in a variety of clinical trials in patients with advanced cancer [47], [48] but cannot be used in the context of vaccine development against pathogens due to their complexity. In addition, multiple injections of α-GalCer have been shown to polarize immune responses and to induce iNKT anergy [49], [50]. Another potential problem for using α-GalCer therapeutically is that α-GalCer displays reduced effectiveness with time after exposure to the pathogen [45]. We therefore decided to use a simple method to test the effect of α-GalCer on the generation of CD8^+^ memory T cells during a well defined murine infection.

Our study showed that treatment with α-GalCer at the time of infection did not affect acute CD8^+^ effector T cell responses. This is consistent with the fact that iNKT cells are dispensable in controlling acute MCMV infection, and therefore should not interfere with mounting an appropriate acute response [28], [51]. Interestingly, as early at 48 hours post-infection, α-GalCer treated mice exhibited decreased splenic and hepatic viral titers compared with vehicle treated controls. Importantly however, α-GalCer treatment did result in a larger MCMV-specific central memory CD8^+^ T cell compartment. Our results are consistent with a recent paper, which shows that α-GalCer boosts memory CTL generation during flu vaccination [16]. In this context, Guillonneau and colleagues found that α-GalCer promoted an increase in the prosurvival gene Bcl-2 and increased long-term survival of activated CD8^+^ T cells [16]. In contrast to the previous study, Bcl-2 levels were not elevated in activated CD8^+^ T cells in the MCMV system (Fig. S3).

During typical MCMV infection, IL-12 induced IFN-γ peaks at 38 hours and other inflammatory cytokines and chemokines including TNF-α, MIP-1α, MCP-1, and IL-6 are present in the blood serum. α-GalCer treatment results in undetectable levels of IL-12 at this time point and significantly decreases levels of other pro-inflammatory signals. It has been determined that for CD8^+^ T cells to be fully activated and expand, they must respond not only to antigen (signal 1) and co-stimulation (signal 2), but also to “signal 3” provided by type I interferon and IL-12 [52], [53]. However, the same inflammatory cytokines promote terminal differentiation and regulate CD8^+^ T cell memory formation through T-bet expression increase [6], [32]. Our data suggest that iNKT cell activation may modulate virus induced inflammatory cytokine production to a sufficient level for signal 3 to be active yet not reaching levels that promote terminal differentiation.

In conclusion, we have shown that α-GalCer treatment enhances the immune response to viral infection, promoting long-term immunity without disrupting the acute effector response. We propose a model in which α-GalCer effects on both the inflammatory response and viral titers drives the antigen CD8^+^ T cell response toward memory differentiation. Interestingly in patients with advanced cancer treated with α-GalCer an increase in CMV specific memory CD8^+^ T cells was observed [54] suggesting that α-GalCer or an α-GalCer analog could also be used therapeutically to control latent infection when the immune system is impaired.

## Materials and Methods

### Mice

C57BL/6 (Taconic Laboratory Animals and Services, Germantown, NY) were purchased for these studies. All mice were maintained in pathogen free facilities at Brown University (Providence, RI). All mice were between 6 and 10 weeks of age at the time of infection.

### Infection and treatment protocols

Stocks of MCMV clone RVG102 (a gift of Dr. Hamilton, Duke University) recombinant for GFP under the Immediate early gene-1 (ie-1) promoter [55] and American Type Culture Collection (ATCC) Strain # VR1399™ were prepared as previously described from salivary glands [55]. Infections were on day 0 with 5×10^4^ (plaque forming units) PFU of MCMV delivered i.p. α-GalCer (Avanti, Japan) or 100 µL vehicle (0.5% polysorbate-20) was delivered 2 µg/mouse i.p. at the same time as MCMV infection.

### Isolation of lymphocytes and blood serum

To obtain splenic lymphocytes, spleens were minced, passed through nylon mesh, layered on lympholyte-M (Cedarlane Laboraties Ltd., Canada), harvested from the interface of the gradient, and washed once in 1% PBS-serum. Hepatic lymphocytes were obtained by mincing and passage through a 70 µm nylon cell strainer (Falcon, Franklin lakes, NJ). Cells were washed 3 times in 1% PBS-serum, and cell suspensions were layered on a two-step discontinuous Percoll gradient (Pharmacia Fine Chemicals, Piscataway, NJ). Fat was removed and lymphocytes were harvested from the interface of the gradient, and washed one time in 1% PBS-Serum. Blood samples were collected by cardiac puncture and spun at 14,000 rpm for 30 min at 4°C. Serum layer was transferred to fresh tube.

### Serum Cytokine Analysis

IFN-γ, IL-4, IL-12p70, TNF-α, MCP-1 (CCL2), IL-6, IL-10, IL-21, GM-CSF, IL-17A and MIP-1α (CCL3) were measured using the cytometric bead array flex sets (BD Pharmingen) and analyzed using FCAP Array Software. The following mAbs were purchased from BD Pharmingen and used for ELISA: IFN-γ mAbs (clone R4-6A2, and clone XMG1.2), IL-4 mAbs (clone 4B11 and BVD6-24G2), and streptavidin-peroxidase. Sandwich ELISA was performed as previously described [15].

### Antibodies and reagents

TCRβ-FITC, CD127-PerCP-Cy5.5, CD62L-PE-Cy7, CD3-APC, CD44-AlexaFluor 750, and CD8-eFluor 450 were purchased from eBioscience (San Diego, CA). M45 tetramer and M57 tetramer were obtained from the National Institute of Allergy and Infectious Disease MHC Tetramer Core Facility at Emory University (Atlanta, GA).

### Flow cytometric analysis

Cells were resuspended in PBS buffer containing 1% FBS. Cells were incubated with 2.4G2 mAb and stained with mAbs specific for cell markers and tetramers for 15 min at 4°C. followed by 15 min at room temperature in the dark. Events were collected on a FACSAria, and the data was analyzed using FlowJo (Tree Star Inc.).

### Determination of viral titers

Organ homogenates were made using the GentleMACS (Miltenyi Biotek) RNA01 program. Standard plaque assay was used to determine viral titers of the organs using MEF cells (Jackson laboratories).

### Statistical analysis

Statistical analysis was done using an unpaired Student's t-test except for Standard plaque assay for which a Mann-Whitney test was performed. Significance was defined as p≤0.05.

### Ethics statement

This study was carried out in strict accordance with the recommendations in the Guide for the Care and Use of Laboratory Animals as defined by the National Institutes of Health (PHS Assurance #A3284-01). Animal protocols were reviewed and approved by the Institutional Animal Care and Use Committee (IACUC) of Brown University. All animals were housed in a centralized and AAALAC-accredited research animal facility that is fully staffed with trained husbandry, technical, and veterinary personnel.

## Supporting Information

Figure S1 **MCMV-specific splenic CD8^+^ T cells display effector phenotype at 7 days post-infection with and without α-GalCer treatment.** Cells were stained with M45 and M57 tetramers in MCMV infected mice (A) and MCMV/α-GalCer mice (B). Tetramer^+^ cells were further characterized using CD62L and KLRG1 (C, D).
(TIF)

Figure S2 **α-GalCer treatment at the time of MCMV infection increases the frequency of IL-7R^+^ MCMV-specific cells at 9 weeks post-infection.** CD8^+^ T cells from mice infected with MCMV or mice treated with α-GalCer at the time of infection were stained with M45 and M57 tetramers (A, B). This population was further characterized with IL-7R (C, D) or CD62L (E, F) and KLRG1.
(TIF)

Figure S3 **α-GalCer treatment does not affect Bcl-2 expression on MCMV-specific CD8^+^ T cells.** CD8^+^ T cells from day 7 MCMV or MCMV/α-GalCer mice were stained with M45 and M57 tetramers, permeabilized, and stained for intracellular Bcl-2 expression. Bcl-2 levels were evaluated on MCMV-specific CD8^+^ T cells with and without α-GalCer treatment. Isotype control is on whole CD8^+^ T cell population.
(TIF)
